# Enhanced lipid metabolism serves as a metabolic vulnerability to polyunsaturated fatty acids in glioblastoma

**DOI:** 10.1172/jci.insight.191465

**Published:** 2025-12-09

**Authors:** Shiva Kant, Yi Zhao, Pravin Kesarwani, Kumari Alka, Jacob F. Oyeniyi, Ghulam Mohammad, Nadia Ashrafi, Stewart F. Graham, C. Ryan Miller, Prakash Chinnaiyan

**Affiliations:** 1 Department of Radiation Oncology, Corewell Health William Beaumont University Hospital, Royal Oak, Michigan, USA.; 2 Department of Metabolomics, Corewell Health Research Institute, Royal Oak, Michigan, USA.; 3 Department of Obstetrics and Gynecology, Oakland University William Beaumont School of Medicine, Rochester, Michigan, USA.; 4 Department of Pathology, Division of Neuropathology, Heersink School of Medicine, University of Alabama at Birmingham, Birmingham, Alabama, USA.; 5 Department of Radiation Oncology, Oakland University William Beaumont School of Medicine, Rochester, Michigan, USA.

**Keywords:** Metabolism, Neuroscience, Oncology, Brain cancer, Metabolomics, Radiation therapy

## Abstract

Enhanced lipid metabolism, which involves the active import, storage, and utilization of fatty acids from the tumor microenvironment, plays a contributory role in malignant glioma transformation, thereby serving as an important gain of function. In this work, through studies initially designed to understand and reconcile possible mechanisms underlying the antitumor activity of a high-fat ketogenic diet, we discovered that this phenotype of enhanced lipid metabolism observed in glioblastoma may also serve as a metabolic vulnerability to diet modification. Specifically, exogenous polyunsaturated fatty acids (PUFAs) demonstrate the unique ability of short-circuiting lipid homeostasis in glioblastoma cells. This leads to lipolysis-mediated lipid droplet breakdown, an accumulation of intracellular free fatty acids, and lipid peroxidation–mediated cytotoxicity, which was potentiated when combined with radiation therapy. Leveraging these data, we formulated a PUFA-rich modified diet that does not require carbohydrate restriction, which would likely improve long-term adherence when compared with a ketogenic diet. The modified PUFA-rich diet demonstrated both antitumor activity and potent synergy when combined with radiation therapy in mouse glioblastoma models. Collectively, this work offers both a mechanistic understanding and a potentially translatable approach of targeting this metabolic phenotype in glioblastoma through diet modification and/or nutritional supplementation that may be readily integrated into clinical practice.

## Abstract 2

Enhanced lipid metabolism in glioblastoma creates vulnerability to polyunsaturated fatty acids, enabling a nutrition-based approach that enhances tumor control and therapeutic synergy with radiation therapy

## Graphical abstract

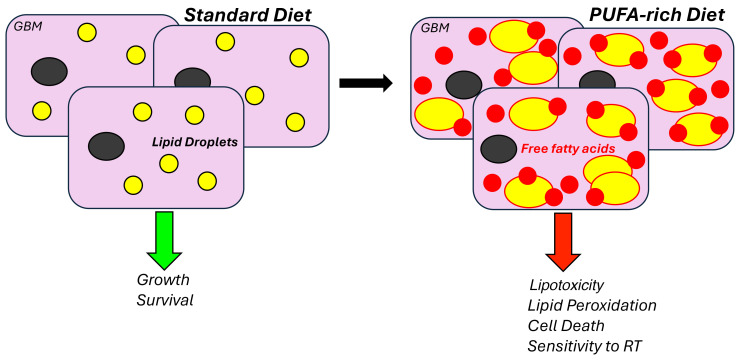

## Introduction

Glioblastoma (GBM) continues to be an invariably fatal malignancy with limited treatment options (1). Despite technological advancements and an unprecedented insight into molecular events that contribute to the growth of these tumors and their immunosuppressive microenvironment, outcomes remain poor, with a median survival of less than 2 years and survival beyond 5 years being uncommon (2). One key factor limiting the clinical advancements observed in other types of cancers to GBM is the blood-brain barrier, which limits a majority of systemic therapies from attaining biologically relevant concentrations in the brain (3). Therefore, additional approaches that can build on current treatment regimens that are effective, well tolerated, and able to evade the blood-brain barrier are needed to improve clinical gains in GBM.

Diet modification and/or nutritional supplementation has gained considerable attention from both the medical community and patients for its potential to prevent or treat a variety of illnesses, including cancer (4, 5), and represents a promising approach to enhance the effectiveness of standard therapies. The ketogenic diet (KD), a high-fat, low-carbohydrate diet, represents one of the most studied approaches. Numerous investigations have demonstrated strong antitumor activity associated with the KD in GBM mouse models, both alone and in combination with radiation therapy (RT) (6–11). Mechanisms underlying this antitumor activity have not been definitively established; however, a majority of studies have attributed it to downstream consequences of the low-carbohydrate aspect of this diet. Others have demonstrated more complexity in the interface between diet modification and its impact on tumor growth; for instance, tumor cell energy homeostasis remained unchanged following a KD (12), and the KD had potential tumor-promoting effects when extended to other models (13). These findings suggest that further studies designed to better understand the interface between diet modification and tumor response are warranted. Clinically, there have been numerous retrospective studies (14–19) and early-phase prospective studies (20–30) evaluating the KD in GBM patients, and ongoing, larger-scale, phase II studies will hopefully provide insight into its potential efficacy (31).

Our group and others have demonstrated that enhanced lipid metabolism plays an important role in GBM pathogenesis at multiple levels, serving as a “gain of function” (32–41). For example, GBM cells have been shown to have the unique ability to actively import fatty acids (40), which are used to provide metabolic plasticity, allowing these cells to adapt to a dynamic microenvironment through fatty acid oxidation (41). However, seemingly paradoxically with regard to these findings, as described above, substantial antitumor activity has been observed in GBM when mice were fed a high-fat/low-carbohydrate KD. Therefore, we performed a series of investigations designed to provide insight into this apparent disconnect. Through these studies, we discovered that the high-fat aspect of the KD plays a contributory role in its observed antitumor activity through dysregulation of lipid homeostasis in GBM cells, independent of carbohydrate restriction. We went on to show that this antitumor activity is primarily attributable to polyunsaturated fatty acids (PUFAs), offering an opportunity to target this metabolic phenotype in GBM through diet and/or nutritional supplementation that may be more readily translated into clinical application.

## Results

### A ketogenic diet demonstrates antitumor activity and enhances RT response in GBM mouse models.

**Figure 1:**
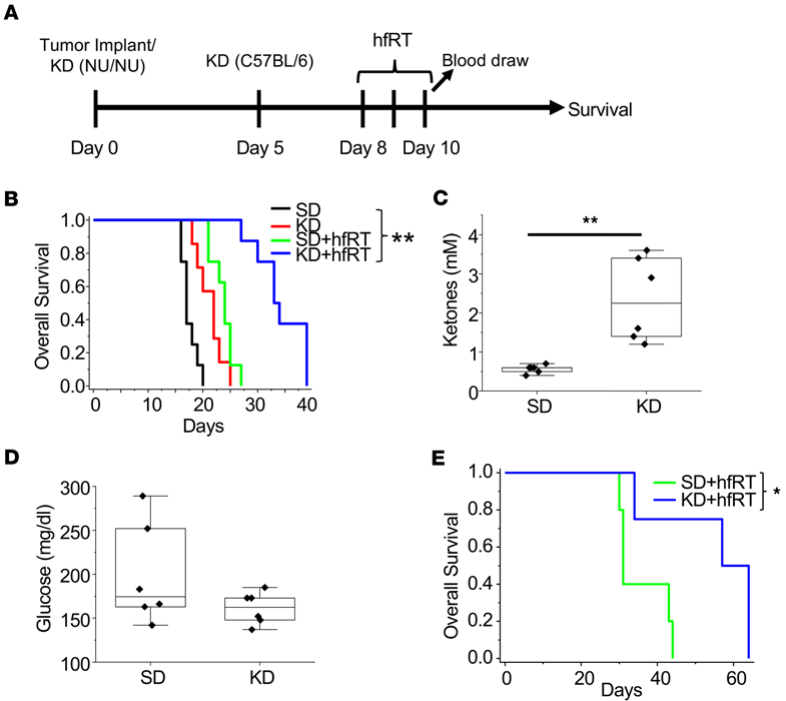
A ketogenic diet demonstrates independent antitumor activity and potent synergy when combined with hypofractionated radiation therapy in GBM. (**A**) Treatment schema. (**B**–**E**) MES83 cells were orthotopically implanted in *nu/nu* mice (*n* = 7–8 mice per arm) (**B**–**D**), and TRP cells were orthotopically implanted in C57BL/6 mice (*n* = 4–5 mice per arm) (**E**) and evaluated for survival and serum markers ketones and glucose after the indicated treatments (*n* = 6 mice per arm). Boxes represent the interquartile range; median and whiskers denote the upper and lower limits. *P < 0.05, **P < 0.005 determined by log-rank test (**B** and **E**) or 2-tailed Student’s *t* test (**C** and **D**). KD, ketogenic diet; hfRT, hypofractionated radiation therapy.

As an initial investigation to begin to reconcile the reported, seemingly paradoxical findings of antitumor activity of a high-fat KD in mouse GBM models in the context of a tumor where enhanced lipid metabolism serves as a gain of function, we evaluated the KD in a patient-derived, GBM tumor–initiating cell line, MES83, that recapitulates the aggressive phenotype of this malignancy (41–43). In these orthotopic studies, mice were either maintained on a standard diet or switched to a KD after tumor implantation, with or without RT (Figure 1A). Irradiated mice were treated on days 8–10 (6 Gy × 3 fractions), and blood was drawn to analyze ketones and glucose on day 10. Consistent with previous reports, mice fed a KD demonstrated improved survival in comparison with standard diet (median survival 17 days vs. 22 days; Figure 1B). Interestingly, the growth delay offered by diet alone was similar to that observed with treatment with a relatively high dose of RT in the context of preclinical models (median survival 23 days). Although an improvement in survival was observed, tumors in these mice continued to grow, with all mice developing neurologic progression by day 27. Although, individually, treatment with RT or a KD alone resulted in a modest increase in survival, the combination demonstrated a dramatic improvement, resulting in a median survival of 33 days with 40% of mice living past 35 days, reiterating the importance of developing combinatorial strategies in this aggressive, molecularly heterogeneous malignancy. As expected, ketosis was observed in mice fed a KD (Figure 1C); however, as in previous publications (24), a significant reduction in serum glucose levels was not observed after 10 days on a KD (Figure 1D). A similar potential to enhance RT response was observed when we extended investigations to the syngeneic GBM mouse line TRP (44–47) (Figure 1E), and the ability of a KD to induce systemic ketosis without altering serum glucose concentrations in mice harboring this line has been previously published (8). Collectively, this supports the concept that mechanisms independent of the low-carbohydrate aspect of the KD may contribute to the observed antitumor activity of a KD and its potential to enhance RT response.

### A ketogenic diet influences intratumoral metabolism in GBM.

**Figure 2:**
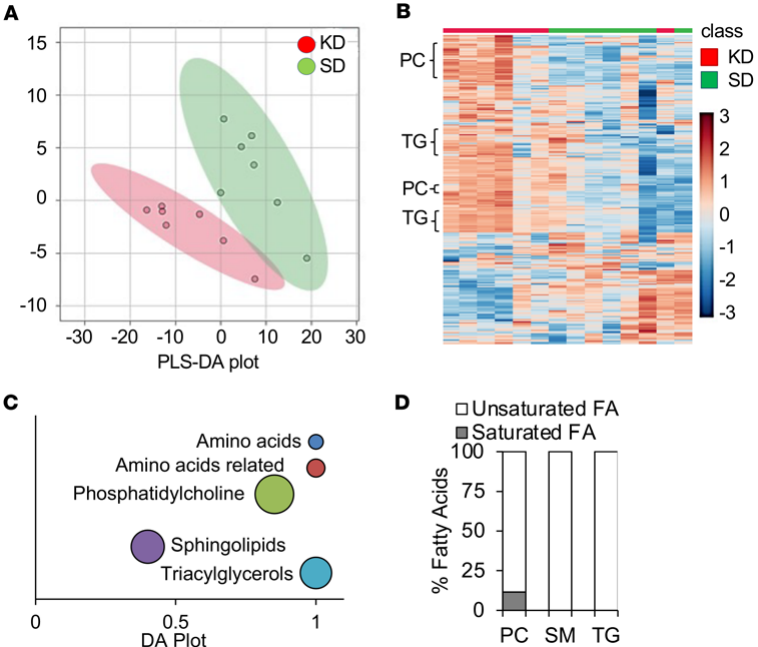
A ketogenic diet modulates intratumoral metabolism in GBM. (**A** and **B**) Global metabolic profiling was performed on MES83 tumors extracted from mice fed a standard diet (SD; *n* = 7) or KD (*n* = 7) and visualized by partial-least-squares discriminant analysis (PLS-DA) score plot (**A**) and hierarchical clustering (**B**). (**C**) Significantly altered metabolites (*P* ≤ 0.05; Wilcoxon’s rank-sum test) in KD-fed mice were classified into major metabolic categories and the differential abundance (DA) scores of each pathway plotted. Metabolic categories having more than 3 metabolites were used in the DA plot. Sizes of the bubbles are relative to the number of metabolites in each category. (**D**) Identified fatty acids for indicated metabolic categories (phosphatidylcholines [PC], sphingomyelins [SM], triacylglycerides [TG]) were classified as saturated or unsaturated fatty acids based on fatty acid chain.

As a KD leads to marked changes in systemic metabolism, we hypothesized that these global changes would influence an individual tumor’s metabolism, potentially serving as a mechanism for its observed antitumor activity. To provide a window into the intratumoral metabolic consequences of a KD in GBM, we performed targeted metabolomic profiling on GBM orthotopic tumors (MES83) from mice fed a standard diet or KD for 10 days. Using the commercially available MxP Quant 500 kit (biocrates life sciences AG) for multiplexed tandem mass spectrometry analysis, a total of 255 metabolites were identified and quantified (Supplemental Table 1; supplemental material available online with this article; https://doi.org/10.1172/jci.insight.191465DS1). Partial-least-squares discriminant analysis indicated that the standard-diet and KD groups were separated across 2 components (Figure 2A), supporting the concept that diet modification can contribute to substantial metabolic changes in an individual tumor. When the metabolomic data were analyzed using unbiased hierarchal clustering by heatmap, clear separation was observed between metabolic profiles from mice fed a standard diet or KD (Figure 2B). Interestingly, tumors from mice fed a KD demonstrated a significant accumulation of lipids, including phosphatidylcholines, sphingolipids, and triacylglycerides, which was recapitulated when plotted using differential abundance scores (Figure 2C). As the accumulation of lipids emerged as a dominant metabolic node in GBM when mice were fed a KD, we went on to evaluate the identified fatty acids in further detail. Intriguingly, when looking at the characteristics of these fatty acids, we observed that an overwhelming majority of these lipids were unsaturated fatty acids (Figure 2D).

### PUFAs modulate lipid dynamics in GBM.

**Figure 3:**
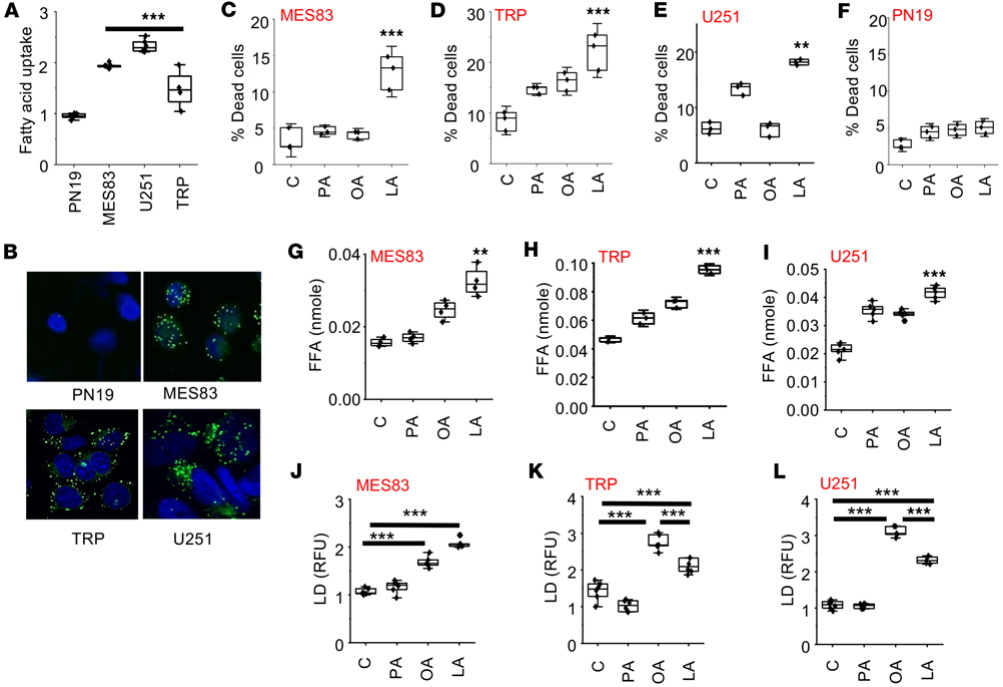
The PUFA linoleic acid modulates lipid homeostasis and induces cytotoxicity in GBM. (**A**) Fatty acid uptake was measured in real time using C1-BODIPY-C12 in the indicated cell lines. (**B**) Cell lines were stained with BODIPY 493/503 and counterstained with DAPI to visualize intracellular lipid droplets using confocal microscopy. Original magnification, ×60. (**C**–**L**) MES83 (**C**, **G**, and **J**), TRP (**D**, **H**, and **K**), U251 (**E**, **I**, and **L**), and PN19 (**F**) cells were treated with indicated fatty acids (palmitate [PA], oleic acid [OA], linoleic acid [LA]; 200 μM). Non-viable cells were counted with trypan blue after 72 hours. Free fatty acids (FFA) and lipid droplets (LD) were measured after 24 hours of the treatment. Boxes represent the interquartile range; median and whiskers denote the upper and lower limits. Data are representative of 3 biologically independent experiments. **P < 0.005, ***P < 0.0005 determined by 1-way ANOVA. RFU, relative fluorescence units. Data presented in **D**–**I** represent a portion of a larger experiment that was performed. These data are re-presented in **B**, **C**, **E**, **F**, **H**, and **I**.

We have previously described the unique capacity of GBM cells to actively import and utilize macronutrients, including lipids, from the microenvironment, a phenotype we termed enhanced metabolic heterotrophy (40). Therefore, we hypothesized that this unique phenotype of GBM cells contributed to the observed intratumoral accumulation of fatty acids in mice fed a high-fat KD and, furthermore, could potentially serve as a metabolic vulnerability. As an initial investigation, we sought to validate this phenotype in our GBM models. Fatty acid uptake was measured in real time (Figure 3A) and visualized using immunofluorescent cytochemistry (Figure 3B) with BODIPY labeling, demonstrating increased uptake in established GBM lines, including the aggressive mesenchymal line MES83, when compared with the proneural GBM line PN19, which we and others have previously shown to recapitulate the phenotype of low-grade glioma (41–43).

We next sought to determine whether this uptake in fatty acids could contribute toward antitumor activity in these GBM lines, recapitulating the observed antitumor activity and lipid accumulation in tumors from KD-fed mice. For these experiments, we tested a panel of fatty acids, including the saturated fatty acid palmitate, the monounsaturated fatty acid (MUFA) oleic acid, and the polyunsaturated fatty acid (PUFA) linoleic acid. Interestingly, in all three GBM lines tested, only the PUFA linoleic acid led to a significant increase in cytotoxicity (Figure 3, C–E). Furthermore, as expected, cytotoxicity was not observed in the proneural line PN19 (Figure 3F), which does not recapitulate the metabolic phenotype of enhanced lipid metabolism/active lipid import observed in GBM. To determine generalizability of the antitumor activity of PUFAs in GBM, we extended investigations to the omega-3 PUFA eicosapentaenoic acid, which demonstrated similar antitumor activity (Supplemental Figure 1). We went on to determine whether the import of exogenous lipids influenced lipid homeostasis in GBM, postulating that these exogenous fatty acids would lead to an accumulation of intracellular free fatty acids, which in turn could contribute toward the observed cytotoxicity in GBM. Consistent with the antitumor activity observed in GBM lines, the PUFA linoleic acid was the only lipid that significantly increased the accumulation of free fatty acids (Figure 3, G–I). Lipid droplets are organelles that play an important role in lipid storage, regulation, and intracellular signaling. Therefore, we examined whether these exogenous fatty acids impacted lipid droplet dynamics in GBM. Although no change in lipid droplet formation was observed with the saturated fatty acid palmitate, both MUFA and PUFA (oleic and linoleic acid, respectively) led to a significant increase in lipid droplet formation (Figure 3, J–L).

**Figure 4:**
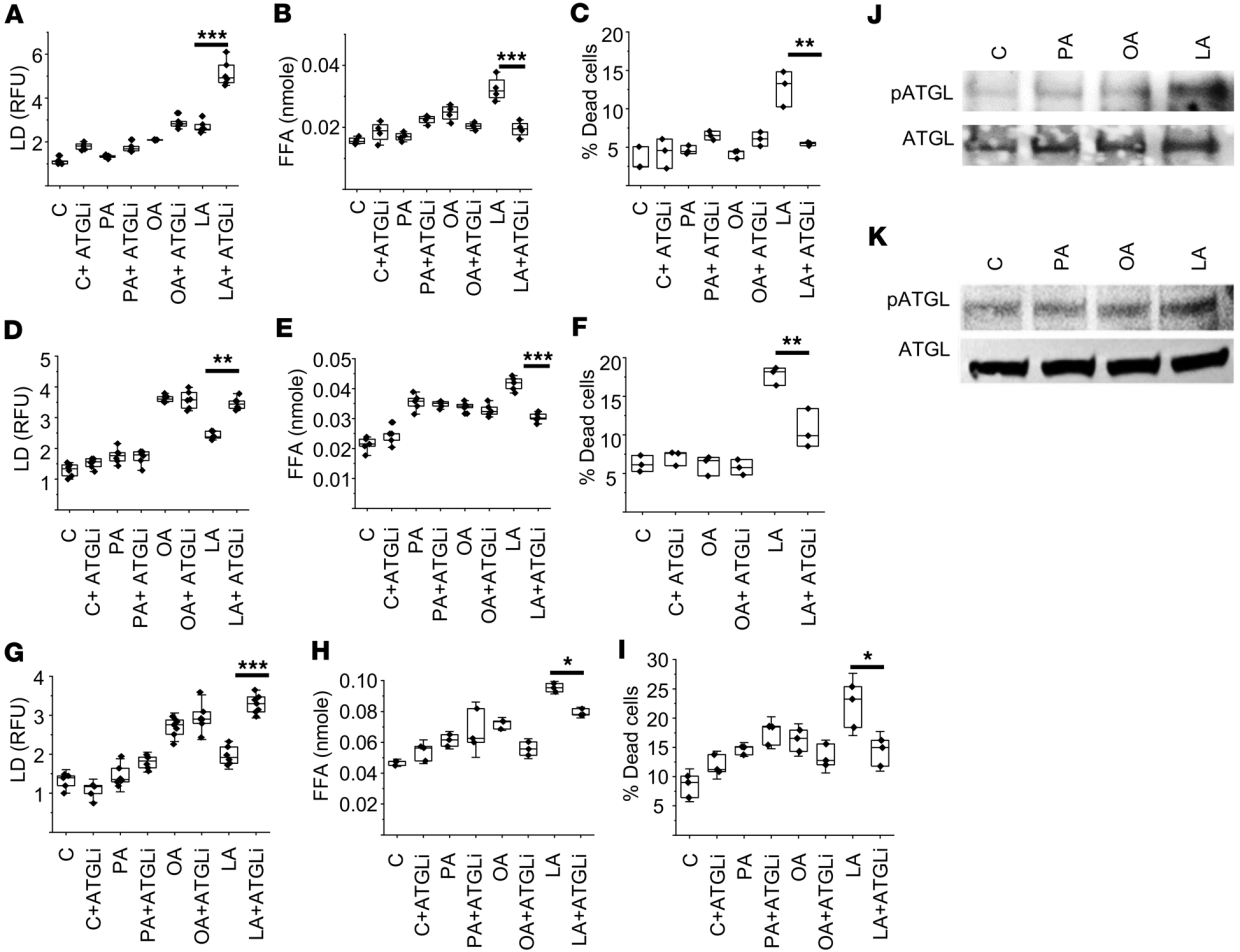
The PUFA linoleic acid modulates lipid droplet dynamics by activating lipase activity in GBM. (**A**–**I**) MES83 (**A**–**C**), U251 (**D**–**F**), and TRP (**G**–**I**) cells were pretreated or not pretreated with atglistatin (ATGLi; 25 μM, 45 minutes) followed by treatment with indicated fatty acids (palmitate [PA], oleic acid [OA], linoleic acid [LA]; 200 μM or control [C]). Lipid droplets (LD; **A**, **D**, and **G**) and free fatty acids (FFA; **B**, **E**, and **H**) were measured after 24 hours of the treatment. Non-viable cells (**C**, **F**, and **I**) were counted with trypan blue after 72 hours. (**J** and **K**) Western blot evaluating indicated proteins and treatment conditions (8 hours) in U251 (**J**) and TRP (**K**) cells. Boxes represent the interquartile range; median and whiskers denote the upper and lower limits. Data are representative of 3 biologically independent experiments. *P < 0.05, **P < 0.005, ***P < 0.0005 determined by 1-way ANOVA. RFU, relative fluorescence units.

Interestingly, in both TRP and U251 lines, there was less lipid droplet formation in the PUFA-treated cells compared with the MUFA group (Figure 3, K and L), which was unique in causing both cytotoxicity and an accumulation of free fatty acids. We therefore explored in further detail how these different classes of fatty acids may differentially modulate lipid droplet dynamics. We postulated that this may be due to dysregulated storage of PUFAs in lipid droplets, resulting in an accumulation of free fatty acids and the observed cytotoxicity with linoleic acid in GBM. Lipolytic pathways play a central role in regulating lipid droplet dynamics and cellular lipid stores (48). Therefore, as an initial investigation, we sought to determine whether exogenous fatty acids could modulate lipase activity in GBM, resulting in the observed changes in lipid droplet dynamics. To test this, we repeated the above-described studies, culturing GBM cells in a panel of fatty acids, with and without the nonspecific lipase inhibitor diethylumbelliferyl phosphate (DEUP) (49). This led to a further increase in lipid droplet formation; however, this was only observed in linoleic acid–treated cells (Supplemental Figure 2, A and B), further supporting the notion that different classes of lipids differentially modulate lipid droplet dynamics in GBM. Studies were extended to determine whether these observed changes in lipid droplet dynamics had biological consequence. Consistent with our running hypothesis, DEUP normalized levels of free fatty acids (Supplemental Figure 2, C and D) and rescued GBM cells from the cytotoxicity of linoleic acid (Supplemental Figure 2, E and F), while having no impact on palmitate- and oleic acid–treated cells. As these studies used the nonspecific lipase inhibitor DEUP, we went on to extend investigations to determine whether the PUFA linoleic acid was directly targeting the lipid droplet by focusing on adipose triglyceride lipase (ATGL), which represents a central enzyme involved in lipid droplet degradation in mammalian cells (50), using the ATGL-specific inhibitor atglistatin (51). Consistent with the above findings, inhibition of the lipase ATGL was specific to the activity of the PUFA linoleic acid, leading to an accumulation of lipid droplets (Figure 4, A, D, and G) and a decrease in intracellular free fatty acids (Figure 4, B, E, and H) and rescuing cells from its antitumor effects (Figure 4, C, F, and I) in all 3 GBM lines. Collectively, these findings support the concept that PUFAs are a unique class of fatty acids that demonstrate antitumor activity in GBM cells, which is mediated by differential modulation of lipid droplet degradation. We went on to evaluate in further detail how PUFAs modulate ATGL activity. Recent studies have demonstrated that phosphorylation of ATGL at Ser(404), which corresponds to murine Ser(406), leads to its activation and lipolysis (52, 53). We therefore went on to determine whether ATGL phosphorylation was influenced by different classes of fatty acids. Indeed, in both human and mouse GBM lines, of the panel of fatty acids tested, only GBM cells treated with the PUFA linoleic acid demonstrated ATGL phosphorylation (Figure 4, J and K), further supporting the direct role PUFAs play in modulating lipid droplet dynamics in GBM. To further validate our proposed mechanism, we extended this line of investigation to the proneural line PN19, which does not recapitulate the phenotype of enhanced lipid metabolism. Consistent with our model, these cells did not demonstrate ATGL phosphorylation or alterations in lipid droplet dynamics when treated with linoleic acid (Supplemental Figure 3).

### Modes of PUFA-induced cell death in GBM cells.

**Figure 5:**
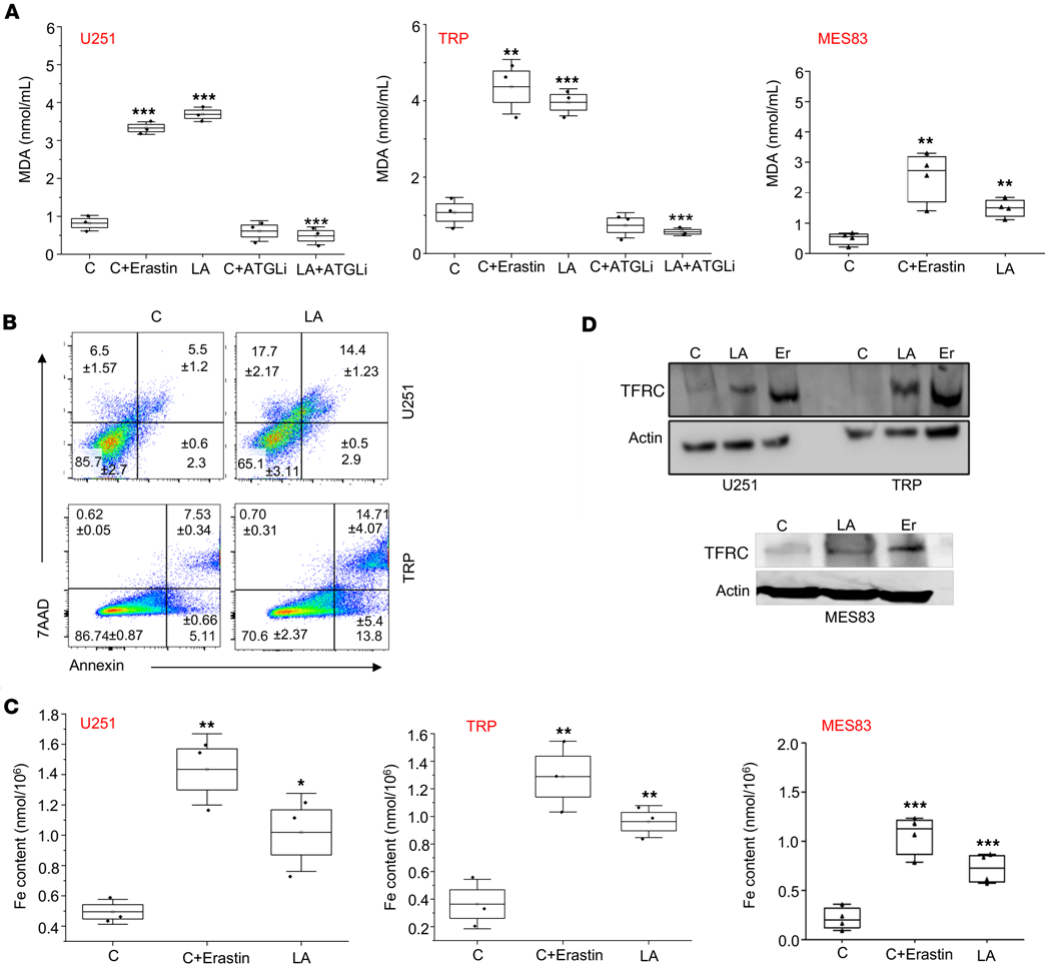
Modes of PUFA-induced cell death in GBM cells. (**A**) Indicated cell lines were treated with vehicle control, erastin (10 μM), linoleic acid (LA; 200 μM), and/or atglistatin (ATGLi; 25 μM) for 24 hours and evaluated for lipid peroxidation using the MDA assay. (**B**) Indicated cell lines were treated with either control or LA (200 μM) for 72 hours and evaluated for apoptosis by annexin V staining using flow cytometry. (**C**) Indicated cell lines were treated with control, erastin (10 μM), or LA (200 μM) for 24 hours and evaluated for ferroptosis by quantification of intracellular iron. (**D**) Western blot evaluating transferrin receptor (TFRC) expression in indicated cell lines treated with control, LA, and erastin (Er). Data are representative of at least 2 biologically independent experiments. *P < 0.05, **P < 0.01, ***P < 0.001 (LA+ATGLi compared with LA) determined by 1-way ANOVA.

We next explored how exogenous PUFAs contributed to the observed cytotoxicity in GBM cells. We demonstrated that treating GBM cells with the PUFA linoleic acid led to an accumulation of free fatty acids, referred to as lipotoxicity (54). Interestingly, of the fatty acids, PUFAs are particularly susceptible to lipid peroxidation based on their unique carbon-carbon double bonds (55), and an increase in peroxidation can trigger multiple modes of cell death, including apoptosis (56) and ferroptosis, a form of iron-dependent cell death (57). We therefore evaluated these pathways in further detail. In all three GBM lines, linoleic acid demonstrated an increase in lipid peroxidation, as measured by the malondialdehyde (MDA) assay, to levels comparable to those of the positive control erastin, which represents an activator of lipid peroxidation and ferroptosis (58) (Figure 5A). Consistent with the above studies demonstrating the ability of lipases to mitigate PUFA-induced accumulation of free fatty acids and cytotoxicity, atglistatin rescued GBM cells from PUFA-mediated induction of lipid peroxidation. We went on to demonstrate multiple modes of cell death contributing to the antitumor activity of PUFA in GBM, including apoptosis as measured by annexin V labeling (Figure 5B), and ferroptosis as measured by iron content and transferrin receptor expression (Figure 5, C and D). Atglistatin rescued linoleic acid–treated GBM cells from ferroptosis, further supporting our proposed mechanism (Supplemental Figure 4).

### Intratumoral heterogeneity of lipid metabolism in GBM.

**Figure 6:**
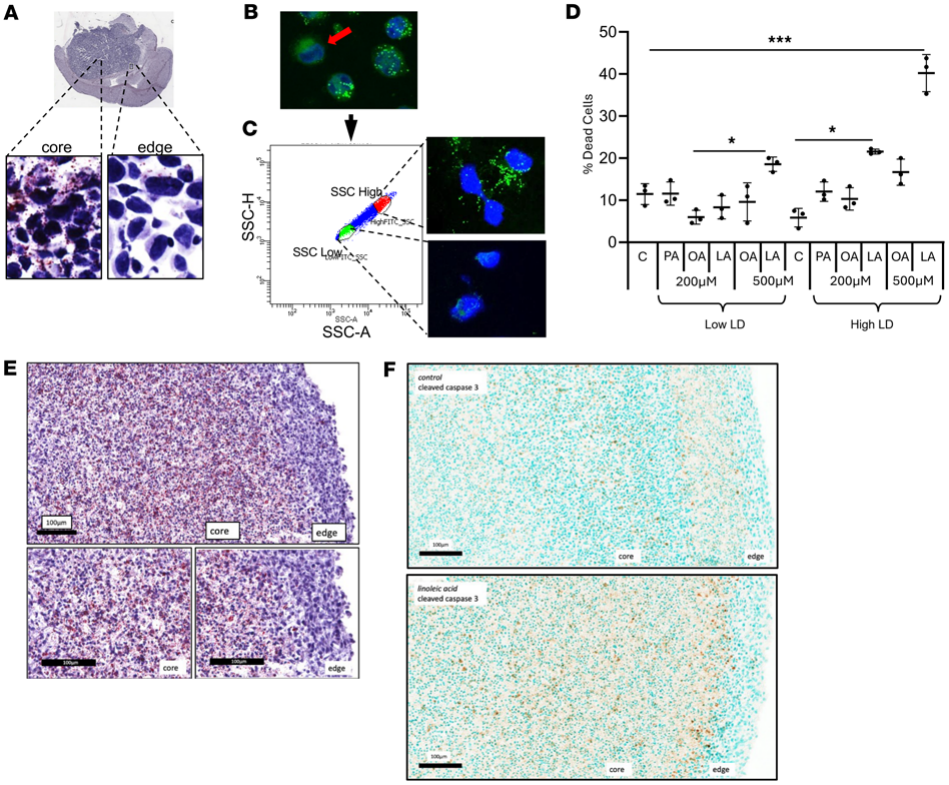
Intratumoral heterogeneity of lipid metabolism in GBM. (**A**) Oil Red O staining was performed on a MES83 tumor grown orthotopically in a mouse to evaluate for the regional accumulation of lipid droplets (LD). Original magnification, ×40. (**B**–**D**) Flow cytometry was used to sort MES83 LD^hi^ and LD^lo^ cells, based on side scattering (**B** and **C**), which were then treated with indicated fatty acids (palmitate [PA], oleic acid [OA], linoleic acid [LA]) at stated concentrations (**D**). Non-viable cells were counted with trypan blue after 96 hours. Original magnification, ×60. (**E**) MES83 cells were grown as organoids for 6 weeks and evaluated for LD using Oil Red O staining. (**F**) MES83 organoids grown for 6 weeks were treated with linoleic acid (200 μM) for 4 weeks and fixed, and immunohistochemical staining for cleaved caspase-3 was performed. Scale bars: 100 μm. *P < 0.05, ***P < 0.0005 determined by 1-way ANOVA. **B** is derived from the same raw image used in Figure 3B and is re-presented here to illustrate LD^hi^ and LD^lo^ cells.

We have demonstrated intertumoral heterogeneity of enhanced lipid metabolism in GBM lines, with this phenotype being present in all lines tested, except the proneural line, which despite being derived from GBM harbors a metabolic phenotype more consistent with a low-grade glioma (40, 42). Interestingly, this molecular subtype has also been shown to be spatially enriched in the peripheral edge of an individual tumor (41, 59). We therefore explored the potential for intratumoral heterogeneity of enhanced lipid metabolism in GBM and sought to determine whether this could influence the observed antitumor activity of PUFAs. Indeed, when we evaluated for lipid droplets in GBM tumors grown orthotopically in mice, we observed lipid droplets predominantly in the “core” of the tumor compared with the “edge” (Figure 6A), further supporting the intratumoral heterogeneity of this phenotype in GBM. To begin understanding the biological consequences of these findings, as an initial investigation, we evaluated for intratumoral heterogeneity in GBM cells grown in culture. Although a majority of cells in culture harbored the metabolic phenotype of enhanced lipid metabolism, which we define as the ability to import fatty acids and store in lipid droplets, we did identify the presence of small populations of cells in culture that did not harbor this phenotype (Figure 6B). We hypothesized that even within this isogenic model, cells harboring this metabolic phenotype would be sensitive to the antitumor activity of PUFAs. To test this, we sorted GBM cells as lipid droplet “high” and “low” by flow cytometry, which was confirmed by immunofluorescent cytochemistry (Figure 6C). We allowed these sorted cells to grow in culture for 48 hours and, after confirming retention of their phenotype, treated them with the panel of fatty acids (Figure 6D). Consistent with our hypothesis, antitumor activity was only observed in lipid droplet–high cells treated with the PUFA linoleic acid. Interestingly, although modest antitumor activity was observed in lipid droplet–low cells treated with a higher dose of linoleic acid (500 μM), a striking increase in cytotoxicity was observed in lipid droplet–high cells.

To allow us to further extend this line of investigation in vitro, we grew a GBM cell line as tumor organoids, which have been previously shown to more accurately recapitulate the tumor microenvironment, including developing regions of central necrosis, along with region-specific differences in lipid metabolism (37). When extending this approach to our model, we observed that GBM organoids recapitulated in vivo findings, with an accumulation of lipid droplets within the “core” compared with the “edge” (Figure 6E). We went on to test the hypothesis that “core” cells harboring a phenotype of enhanced lipid metabolism would be particularly sensitive to the antitumor activity of the PUFA linoleic acid. Indeed, cleaved caspase activity was spatially enriched to the “core” of organoids cultured with linoleic acid when compared with the “edge” (Figure 6F). Collectively, our findings demonstrate that there is clear intratumoral heterogeneity of enhanced lipid metabolism in GBM and that this phenotype may serve as metabolic vulnerability to PUFA-mediated cytotoxicity.

### Rational combinatorial strategies to enhance PUFA-mediated cytotoxicity in GBM.

**Figure 7:**
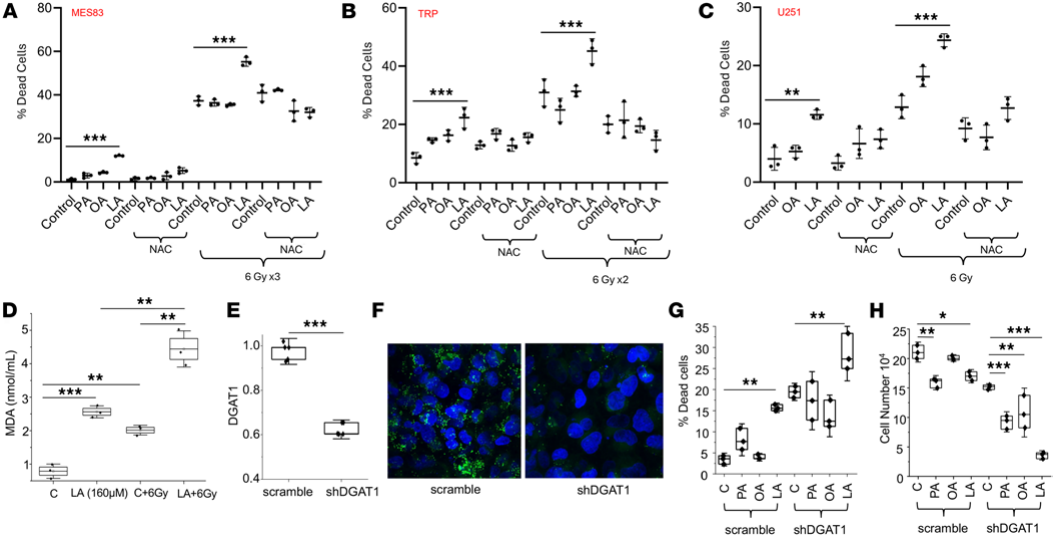
Combinatorial strategies to enhance PUFA-mediated cytotoxicity in GBM. (**A**–**C**) Indicated cell lines were treated with fatty acids (palmitate [PA], oleic acid [OA], linoleic acid [LA]; 200 μM) alone or in combination with *N*-acetylcysteine (NAC; 1 mM) with and without radiation at stated dose. Non-viable cells were counted with trypan blue after 72–96 hours. (**D**) U251 cells were treated with either vehicle control or linoleic acid (LA; 160 μM) for 24 hours with and without RT (6 Gy) and evaluated for lipid peroxidation using the MDA assay. (**E** and **F**) Knockdown of DGAT1 expression in U251 cells using shRNA was validated using RT-PCR (**E**), and cells were evaluated for intracellular lipid droplets with BODIPY 493/503 using confocal microscopy (**F**). Original magnification, ×60. (**G** and **H**) Antitumor activity of the indicated fatty acids was evaluated in shRNA scramble control shDGAT1 U251 cells. Viable and non-viable cells were determined by trypan blue after 72 hours. *P < 0.05, **P < 0.005, ***P < 0.0005 by 1-way ANOVA (**A**–**D**, **G**, and **H**) or 2-tailed Student’s *t* test (**E**). Data presented in **B** without NAC and/or RT are derived from data presented in Figure 4I.

Although we demonstrated antitumor activity of the PUFA linoleic acid in our panel of GBM cell lines, the observed activity was modest as a single agent. We therefore explored rational combinatorial strategies that may be utilized to potentiate this activity. As an initial investigation, we sought to recapitulate our in vivo data, which demonstrated strong synergy between the KD and RT. As demonstrated previously, antitumor activity was observed with the PUFA linoleic acid alone, and, as expected, irradiating MES83 GBM cells alone led to robust cytotoxicity (Figure 7A). As hypothesized and consistent with our previous findings, of the panel of fatty acids tested, only the PUFA linoleic acid demonstrated the capacity to potentiate the antitumor activity of RT. Similar findings were observed when we extended this line of investigation to TRP and U251 cells (Figure 7, B and C). We have shown that PUFAs lead to lipotoxicity and lipid peroxidation in GBM cells, which was rescued by the lipid droplet lipase inhibitor atglistatin. We hypothesized that oxidative stress plays a contributory role in both the observed antitumor activity mediated by the accumulation of these free fatty acids in GBM and the potent synergy when combined with RT. To test this, we performed combination studies with the antioxidant *N*-acetylcysteine (NAC). In all 3 cell lines tested, NAC rescued cells from both the independent activity of the PUFA linoleic acid and when combined with RT (Figure 7, A–C).

As described above, PUFAs are particularly susceptible to free radical–induced lipid peroxidation based on their unique carbon-carbon double bonds. As the antitumor activity of RT primarily involves the generation of free radicals, we hypothesized that this combination could contribute toward the observed synergy between RT and PUFAs. Using a slightly lower concentration of linoleic acid to more clearly demonstrate an interaction, the combination of linoleic acid and RT resulted in a significant increase in lipid peroxidation (Figure 7D). Collectively, these findings support the concept that PUFA-mediated dysregulation of lipid metabolism results in antitumor activity in GBM cells, both alone and in combination with RT, through oxidative stress.

Next, as another strategy to enhance PUFA-mediated antitumor activity in GBM, we examined combinatorial approaches designed to modulate lipid droplet dynamics. Specifically, it has recently been shown that the storage of fatty acids into lipid droplets is regulated by the protein diacylglycerol acyltransferase 1 (DGAT1) in GBM cells, and its inhibition disrupted lipid homeostasis, resulting in high levels of reactive oxygen species and apoptosis (35). We therefore hypothesized that a cell’s inability to efficiently store fatty acids could render it more susceptible to the antitumor activity of exogenous PUFAs. Consistent with previous work, shRNA knockdown of DGAT1 in U251 cells inhibited lipid droplet formation (Figure 7, E and F) and demonstrated antitumor activity (Figure 7, G and H). The combination of shRNA knockdown of DGAT1 and only exogenous linoleic acid led to an additive increase in cytotoxicity (Figure 7G). Interestingly, when we evaluated for cellular proliferation, there appeared to be varying degrees of antiproliferative effects in the fatty acids tested; however, the combination of shRNA knockdown of DGAT1 and exogenous linoleic acid led to a profound reduction in proliferation (Figure 7H). This supports the concept that rational combinations designed to target lipid droplet homeostasis may enhance therapeutic response in GBM.

### Evaluating the antitumor activity of a PUFA-rich diet in GBM in vivo.

**Figure 8:**
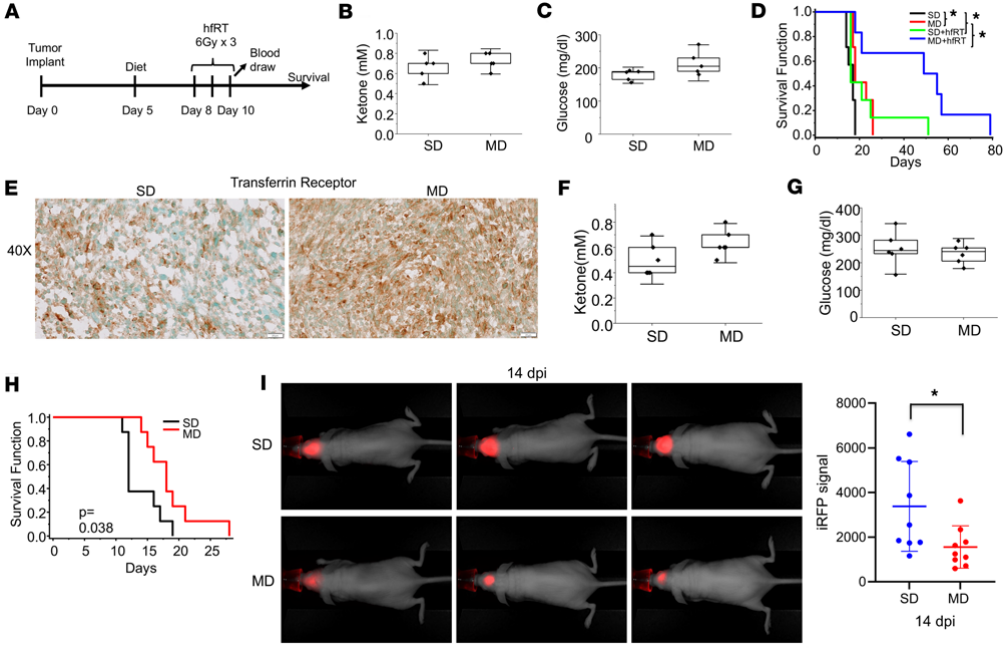
Evaluating the antitumor activity of a PUFA-rich diet in GBM in vivo. (**A**) Treatment schema. (**B**–**D**) U251 (*n* = 6–7 per group) tumors were grown intracranially in *nu/nu* mice and treated according to the schema. After the tumor implantation, mice were fed either with a standard diet (SD) or a PUFA-rich modified diet (MD) followed by treatment with 6 Gy of radiation on days 8, 9, and 10 post-implantation (hypofractionated radiation therapy [hfRT]) as described in the schema. (**E**–**H**) To validate the antitumor activity of a PUFA-rich diet, we extended investigations to TRP tumors (*n* = 8 per group) grown intracranially in C57BL/6 mice and fed either SD or MD. (**E**) Representative images of transferrin receptor immunohistochemistry staining in TRP xenograft tumor tissue from mice fed SD or MD. Scale bars: 20 µm. (**F** and **G**) Blood ketone and glucose concentrations. (**H**) Survival of C57BL/6 mice (*n* = 8) fed SD or MD. (**I**) In vivo fluorescence imaging was performed on *nu/nu* mice bearing MES83-iRFP tumors (*n* = 9) at 14 days post-implantation (dpi). Left panel: The fluorescent signal (red) is more intense in the SD group (top) compared with the MD group (bottom). Right panel: Quantification of iRFP signal. Blood ketone and glucose concentrations were measured using the Precision Xtra meter (**B** and **C**, *n* = 5 per group; **F** and **G**, *n* = 6 per group). *P < 0.05. Statistical analyses by 2-tailed Student’s *t* test (**B**, **C**, **F**, **G**, and **I**) or log-rank test (**D** and **H**).

In the work presented thus far, although both the antitumor activity of a KD and its capacity to potentiate RT response were confirmed in our GBM models, correlative studies coupled with systematic characterization of lipid droplet dynamics suggest that the high concentrations of fatty acids in this diet (namely PUFAs) play a contributory role in the observed antitumor activity. To extend this line of investigation in vivo, we designed a modified PUFA-rich diet (mPD) formulated to exploit the observed antitumor activity of these fatty acids in GBM. Importantly, without requiring carbohydrate restriction, this diet will likely improve tolerability and clinical implementation of this approach. Specifically, the components of this more balanced diet consisted of 60%, 30%, and 10% kcal from fat, protein, and carbohydrates, respectively, compared with 90%, 9%, and 1% in a KD. Fat sources rich in PUFAs were used to prepare the modified diet, which constituted the majority of fat (1.92 kcal/g), followed by monounsaturated (0.705 kcal/g) and saturated fatty acids (0.341 kcal/g). Furthermore, the n6:n3 ratio in the mPD was within the recommended range of 1:1 to 3:1 (60). We therefore tested this PUFA-rich diet in our GBM models (Figure 8A). The diet was well tolerated, and there was no change in body weight, liver function, or renal function in mice in comparison with standard diet and/or KD (Supplemental Figure 5). As expected, there were no changes in systemic ketosis or glucose concentrations in serum from mice fed an mPD compared with a standard diet (Figure 8, B and C). Consistent with our hypothesis, the mPD demonstrated both antitumor activity and potent enhancement of RT response in U251 tumors (Figure 8D). In addition, we validated the potential of a PUFA-rich diet to induce ferroptosis in vivo in GBM (Figure 8E). We went on to validate the antitumor activity of this diet in the TRP (Figure 8, F–H) and MES83 (Figure 8I) lines. Collectively, this work supports the concept that the phenotype of enhanced lipid metabolism observed in GBM may serve as a metabolic vulnerability that may be therapeutically exploited by a PUFA-rich diet and/or supplementation.

## Discussion

Diet modification has garnered considerable enthusiasm in the scientific community as a strategy to complement traditional cancer therapies to improve clinical outcomes (61). In addition, this approach has resonated with patients, as it provides them with an opportunity to play a more active role in their cancer journey. This is particularly relevant in GBM, as scientific advancements have made limited gains in improving outcomes and diet modification could potentially circumvent the blood-brain barrier, which represents a considerable impediment to many, otherwise promising, therapeutics. Despite this level of enthusiasm, diet modification has yet to be deemed a “standard of care” in the management of any type of cancer. For GBM specifically, a majority of research involving diet modification has focused on the KD. Thus far, clinical testing has largely involved relatively small pilot studies; however, an ongoing, larger-scale phase II study may finally provide insight into its potential efficacy in GBM (31). The wide-ranging adoption of diet modification as a bona fide treatment strategy in cancer, and its rigorous, prospective testing in the context of clinical trials, have been hindered by several factors. One factor is a limited understanding of underlying mechanisms contributing to the potential antitumor activity of a specific diet. This is further exacerbated by a lack of appreciation of the complex interface between systemic metabolism and the biology of an individual tumor, leading to an oversimplified perception of diet impacting a wide array of molecularly diverse types of cancers in a similar way. In GBM, several preclinical investigations have demonstrated potent antitumor activity of a KD (6–11), yet mechanistic underpinnings have yet to be clearly defined and are likely multifactorial. In terms of mechanism(s) of antitumor activity, a major emphasis has been on the low-carbohydrate aspect of this diet, which has often been dogmatically associated with “starving” a highly glycolytic tumor. As our study and others have shown that serum glucose levels normalize quickly after initiation of this diet, this perceived lack of glucose as a primary mechanism of antitumor activity is unlikely. However, other factors associated with carbohydrate restriction, including systemic ketosis and/or insulin growth factor signaling, may play a contributory role.

Our work, presented herein, was stimulated by metabolomic profiling of GBM tumors from KD-fed mice, which demonstrated considerable metabolic changes within these tumors, most notably an accumulation of PUFAs. Based on these results, we designed a series of experiments to determine whether the high-fat aspect of a KD could independently play a contributory role in the observed preclinical activity of this diet. This line of investigation is supported by our previous work, which demonstrated the unique ability of GBM cells to actively import macronutrients from the microenvironment, including fatty acids and proteins, and utilize these substrates for survival and/or growth, a process we termed enhanced metabolic heterotrophy (40). Although this metabolic phenotype appears to serve as a gain of function in GBM cells, we postulated that it evolved in the context of a normal diet, and therefore does not require parallel molecular machinery to be in place to actively regulate this process. As it is well recognized that an overload of fatty acids can short-circuit intracellular lipid storage homeostasis, leading to disrupted cellular functions and lipotoxicity (54), we hypothesized that this unique phenotype observed in GBM cells may be exploited, serving as a metabolic vulnerability to a high-fat diet. Consistent with this hypothesis, exogenous fatty acids induced cytotoxicity in GBM cells harboring this metabolic phenotype. Interestingly, when tested using a panel of fatty acids, including the saturated fatty acid palmitate, the monounsaturated fatty acid oleic acid, and the PUFA linoleic acid, antitumor activity in GBM cells, which resulted in an accumulation of free fatty acids and increase in lipid peroxidation, was specific to PUFAs. Our findings are consistent with a recent study by Dierge et al., which attributed these findings to increased lipid peroxidation induced by the acidic microenvironment in cancer (62). We went on to identify the lipid droplet–specific lipase ATGL, through phosphorylation-mediated activation, as the primary target of PUFAs, resulting in the observed accumulation of the observed free fatty acids.

Using a GBM organoid model (32–41), we identified intratumoral heterogeneity of this metabolic phenotype in GBM, along with its therapeutic implications. This line of investigation is supported by our previous work, which identified this phenotype as being a requisite adaptation that enables GBM cells to acclimate to their dynamic microenvironment (41), and the observation that the accumulation of lipid droplets in GBM organoids is regionally confined to the peritumoral core of an individual tumor (37), which was validated in our mouse model. Specifically, the tumor core represents a nutrient-deprived region, where GBM cells likely rely on the active import of macromolecules from the microenvironment to serve as metabolic substrates. Alternatively, PUFAs’ unique chemical structure, consisting of carbon-carbon double bonds, renders them particularly susceptible to free radical–induced lipid peroxidation, which may be particularly relevant in the core region of a tumor. Therefore, the regional accumulation of lipid droplets may serve as a protective niche in GBM cells, diverting these reactive fatty acids away from cell membranes, making them less vulnerable to peroxidation and ferroptosis-mediated cell death (63–65). In either model, our work suggests that this metabolic adaptation may serve as an exploitable vulnerability and framework to design rational combinatorial treatment regimens to target intratumoral heterogeneity in GBM.

In addition to a sound understanding of the mechanistic underpinnings contributing toward the antitumor activity of a specific diet in a specific tumor, another factor hindering the successful translation of diet modification into clinical application is long-term adherence to a particular diet. For example, in epilepsy patients, the mean adherence rate of adults to a KD was 63.9% and dropped to 37.7% at 36 months (66). Similar challenges of adherence to a KD were observed when it was tested in a type 2 diabetes population (67). Furthermore, there is a lack of systematic methods for monitoring adherence to a particular diet, which would be critical for evaluating efficacy of diet modification in the context of clinical trials. One can speculate that these challenges would be further intensified in GBM patients, who are often on corticosteroids to manage peritumoral edema, leading to common side effects of increased appetite and elevated blood glucose levels. As our mechanistically based work identified direct antitumor activity of PUFAs in GBM, we hypothesized that a PUFA-rich diet could have antitumor activity independent of carbohydrate restriction and/or systemic ketosis in mice, which could facilitate adherence. To test this, we developed a PUFA-rich diet formulation. Although the total percentage of kilocalories from fat in this modified diet was about 60%, compared with about 90% in the KD, PUFAs made up the majority of these fatty acids and were comparable to the quantity of PUFAs in the KD. This modified diet allowed for an approximately 7-fold increase in carbohydrates and, as expected, did not lead to systemic ketosis. Consistent with our hypothesis, a modified, PUFA-rich diet demonstrated both independent antitumor activity and potent ability to enhance RT response in GBM mouse models. Intriguingly, in stark contrast to traditional cytotoxic chemotherapies, PUFAs have been associated with numerous health benefits. These include improved physical functioning, general metabolism, and heart health in the context of cardiovascular disease (68, 69), making the integration of PUFAs into combinatorial strategies in the treatment of GBM particularly attractive. Although only diet modification was tested here, these findings provide a framework for alternative approaches, included PUFA supplementation using more conventional approaches (e.g., fish or flaxseed oil pills) rather than modifying an entire diet, which we anticipate would aid in both adherence and, being more readily quantifiable, translatability for systemic testing in the context of clinical trials.

## Methods

### Sex as biological variable.

Our in vivo experiments were conducted using female mice. Female nude mice were used in our orthotopic GBM experiments because they exhibit markedly lower levels of aggression compared with male mice. Reduced aggressiveness is important in group‑housed conditions, as it minimizes fighting‑related injuries during handling or pooling of animals — events that can introduce biological variability and confound experimental outcomes (70). The findings are based on the established biological mechanisms explored and are not expected to differ based on sex.

### Cell lines.

MES83, MES83-iRFP, and PN19 cells (provided by Ichiro Nakano, Heersink School of Medicine, University of Alabama at Birmingham, Birmingham, Alabama, USA) were cultured in neurosphere complete medium DMEM/F12, GlutaMAX (Gibco) medium supplemented with 2% (vol/vol) B-27 (Gibco), 20 ng/mL human-rFGF (PeproTech), 0.25% (vol/vol) heparin (STEMCELL Technologies Inc.), and 20 ng/mL human-rEGF (PeproTech). TRP cells were generated as previously described (44, 47), and grown in MEM supplemented with MEM non*-*essential amino acids (Gibco) and 10% FBS (Gibco) (44, 71). U251 cells were purchased from ATCC and were grown in DMEM (Corning) supplemented with 10% FBS. HEK293T cells were grown in DMEM supplemented with 10% FBS and GlutaMAX (Gibco). Cell viability was assessed using the trypan blue dye exclusion test. For in vitro radiation studies, cells were treated in a Cabinet Irradiator (Xstrahl Inc.).

### Reagents.

Ten percent bovine serum albumin (BSA) in DPBS, BSA-conjugated oleic and linoleic acid, diethylumbelliferyl phosphate (DEUP), *N*-acetylcysteine (NAC), and atglistatin were purchased from Sigma-Aldrich. BSA and BSA-conjugated palmitate were obtained from Cayman Chemical.

### Animal handling.

Orthotopic xenografts of MES83, MES83-iRFP, and U251 tumors were established in female *nu/nu* mice (Charles River Laboratories) using previously described methods (46). TRP tumors were orthotopically established in C57BL/6 mice (The Jackson Laboratory). Radiation was administered using the Faxitron (Faxitron X-Ray Corp.) with custom lead shield designed to shield the remaining body (46). Survival was analyzed using Kaplan-Meier curves. Mice were euthanized upon reaching the endpoint criteria, which included neurologic signs and/or morbidity.

### Diet.

All animals in all experiments were fed ad libitum. Food was monitored and changed daily. The compositions of the diets were as follows:

The control group was maintained on a standard mouse chow diet (Teklad 2020x for C57BL/6 and Teklad 2020sx for *nu/nu* mice).

Ketogenic diet with a 4:1 ratio was purchased from Envigo or Bioserve. The sources of fat in the ketogenic diet were lard, anhydrous milk fat, and corn oil and included the following nutrients, protein (8.4% kcal), carbohydrate (1.4% kcal), and fat (90.3% kcal), and fats, PUFA (1.3 kcal/g), MUFA (2.4 kcal/g), and saturated (2.74 kcal/g).

PUFA-rich modified diet was purchased from Envigo. Corn and flaxseed oil were used as fat sources to prepare the custom-modified diet and included the following nutrients, protein (29.9% kcal), carbohydrate (10.0% kcal), and fat (60.0% kcal), and fats, PUFA (1.92 kcal/g), MUFA (0.705 kcal/g), and saturated (0.341 kcal/g).

### Blood ketone and glucose analysis.

Serum ketone and glucose levels were measured using the Precision Xtra blood and ketone monitoring system, following the manufacturer’s instructions.

### In vivo optical imaging.

A Pearl Impulse Small Animal Imaging System (LI-COR) was used for in vivo imaging of the index lesion 14 days after implantation of MES83-iRFP cells. Mice were anesthetized using isoflurane/oxygen during the imaging process. All images were acquired with the same parameter settings. The RFP signal was determined with Image Studio Software (v5.2, LI-COR).

### Metabolomic profiling and data analysis.

Metabolites were analyzed using the MxP Quant 500 kit as directed in the manufacturer’s instructions (biocrates life sciences AG). Briefly, tissue was lyophilized, extracted using 100% isopropanol, homogenized, and centrifuged to collect the extracted supernatant. Samples and standards were included in a premix of phenylisothiocyanate for derivatization and subsequently dried under nitrogen. Samples were extracted in 5 mM ammonium acetate in methanol, and the extracts were collected by centrifuging of the preparation plate. Sample extracts were diluted with H_2_O (1:1) for the liquid chromatography phase of the analysis. For the flow injection analysis (FIA), the sample extract was mixed with the kit solvent in a separate plate, and the QC sample extract was mixed with the kit solvent. Sample extracts were analyzed using a Waters I-class UPLC unit coupled with a Waters Xevo-TQ-S (Waters Corp.). For UPLC analysis, sample extracts were separated using the MxP Quant 500 C18 column with an attached guard and precolumn mixer (biocrates life sciences AG). The mobile phase consisted of A: H_2_O and formic acid (0.2%) and B: MeCN and 4-formic acid (0.2%) delivered at a flow rate of 0.8 mL/min with a gradient of B: 0%–100% over 4.50 minutes. Eluent %B was increased to 1.00 mL/min flow rate and maintained at 100% for 30 seconds, followed by a rapid return to the initial conditions for 70 seconds to equilibrate the column. Both positive- and negative-mode gradients were 5.80 minutes long. The negative-mode acquisition gradient differed from the positive-mode with a difference in %B composition between 2.00 and 4.50 minutes. The injection volume was 5 μL for positive data acquisition and 15 μL for the negative run. The wash solvent composition consisted of H_2_O/MeOH/MeCN/isopropanol (vol/vol). The Quant 500 Kit offers direct flow injections (FIA) for lipid analysis. An isocratic method was performed using the kit-provided solvent (290 mL MeOH: 1 ampule of FIA additives). The isocratic mobile phase (B: 100% MeOH) was delivered at a low flow rate of 0.03 mL/min. The injection volume was 20 μL for both positive- and negative-mode acquisitions. All data were extracted using MetIDQ software (biocrates life sciences AG). Metabolites that were below the limit of detection in all treatment conditions or were identified in less than 50% of samples in a group were not reported. The differential abundance score was calculated as described previously (40). Heatmap and partial-least-squares discriminant analysis were generated using MetaboAnalyst (72).

### Fatty acid uptake.

Evaluation of fatty acid uptake was performed as previously described (40). Briefly, cells were cultured in a 96-well plate overnight, and medium was replaced with HBSS medium. The cells were then incubated for 1 hour in a humidified 37°C incubator with 5% CO_2_. A mixture of fatty acid–BSA BODIPY (5 mM HEPES, 10 mM BODIPY, 5 mM fat-free BSA, and 4 mM trypan blue) was added to the cells, and fluorescence was measured at excitation and emission wavelengths of 485 and 528, respectively, using a SpectraMax Gemini EM Microplate Reader (Molecular Devices).

### Confocal microscopy analysis of lipid droplets.

Cells were stained with BODIPY 493/503 (0.5 μM) for 15 minutes followed by counterstaining with DAPI (Bio-Rad) and visualized by confocal microscopy (Nikon Eclipse Ti, ×40 or ×60 oil). Images were processed using Fiji software (NIH), as previously described (35, 73).

### Free fatty acid analysis.

Intracellular free fatty acids were analyzed using the Free Fatty Acid Quantitation Kit (Sigma-Aldrich) per the manufacturer’s instructions. Briefly, cells were homogenized in a 1% (wt/vol) Triton X-100 (EMD Chemicals) in chloroform solution (Sigma-Aldrich), followed by centrifugation at 13,000*g* for 10 minutes to remove insoluble material. The organic phase (lower phase) was collected and air-dried at 50°C to remove chloroform, followed by vacuum drying for 30 minutes to remove any trace chloroform. The dried lipids were then dissolved in fatty acid assay buffer by extensive vortexing for 5 minutes. Free fatty acids were quantified using colorimetric assay methods with an XMARK microplate spectrophotometer (Bio-Rad).

### Flow cytometric analysis of lipid droplets and apoptosis.

Cells were stained with BODIPY 493/503 (1 μM) to evaluate lipid droplets (74). The samples were then analyzed using a BD FACSCanto II flow cytometer (Becton Dickinson), and the analysis was carried out using FlowJo v10 Software (FlowJo LLC). Apoptotic cells were assessed through staining with annexin V (Invitrogen) and 7AAD (Calbiochem) per the manufacturer’s protocol.

### Western blot.

Western blot was performed using methods previously described (8). A custom antibody against pATGL was generated by ABclonal. Antibody against ATGL was purchased from Cell Signaling Technology (no. 2138). Antibody against phosphorylated ATGL was obtained from Abcam (ab135093). Antibody against TFRC was obtained from Abcam (ab214039). HRP-conjugated secondary antibodies were obtained from Sigma-Aldrich (13-348 and 12-349).

### Lipid peroxidation.

Lipid peroxidation was determined by a malondialdehyde (MDA) assay kit (ab118970, Abcam). Briefly, cell supernatants after homogenization were mixed with thiobarbituric acid and heated for 1 hour at 95°C. After cooling on ice, the absorbance of the samples was measured at 532 nm. The content of MDA was calculated by a standard concentration curve.

### Ferroptosis.

Ferroptosis was determined by iron content using an iron assay kit (ab83366, Abcam). Briefly, cells were collected, homogenized (in cold iron assay buffer), and centrifuged. Five microliters of iron reducer was added to samples, which were incubated for 30 minutes at 37°C. Next, 100 μL of iron probe was added, and samples were incubated for 60 minutes at 37°C in the dark. The absorbance at 593 nm was measured using a microplate reader.

### Tumor organoids.

Tumor organoids were cultured using methods previously described (37). Briefly, we created GBM organoids by suspending 10,000 tumor cells in Matrigel and forming 20 μL pearls on an Organoid Embedding Sheet (STEMCELL Technologies Inc.), before culturing them in 6-well or 10 cm plates with shaking in neurosphere complete medium.

### Tissue processing.

Mouse brains and organoids were immersed in 4% (wt/vol) paraformaldehyde overnight for fixation, cryoprotected in 30% sucrose, embedded in OCT compound, and sectioned at a thickness of 10 μm using a cryostat.

### Oil Red O staining.

Lipid droplets in tumors and tumor organoids were measured by Oil Red O staining. Frozen sections were air-dried at room temperature, washed with PBS, rinsed with 60% isopropanol, and stained with Oil Red O working solution (freshly prepared from 60 mL of Oil Red O solution from Sigma-Aldrich and 40 mL distilled water) for 15 minutes. After staining, slides were rinsed with 60% isopropanol, followed by 2 rinses of distilled water, counterstained with Modified Mayer’s Hematoxylin (Abcam), rinsed in tap water, and mounted in aqueous mounting medium (Newcomer Supply). Sections were visualized under Leica Aperio.

### Immunohistochemistry.

Frozen sections were air-dried at room temperature, washed in PBS, and then subjected to antigen retrieval in 0.1 M citrate buffer (pH 6; Vector Laboratories) for 10 minutes using a steamer. Sections were incubated with 0.3% H_2_O_2_ in methanol for 30 minutes to block endogenous peroxidase activity. After blocking with PBS containing 5% normal goat serum (Vector Laboratories) and 0.3% Triton X-100 (Calbiochem) for 1 hour, sections were incubated with primary antibody (cleaved caspase-3, Cell Signaling Technology 9661; transferrin receptor, Abcam 214039) diluted in Antibody Diluent Reagent Solution (Life Technologies) overnight at 4°C. Sections were washed with PBS and incubated with SignalStain Boost IHC Detection Reagent (Cell Signaling Technology) for 1 hour at room temperature. DAB (Vector Laboratories) was used as a chromogen, and sections were counterstained using methyl green (Vector Laboratories). Sections were visualized under Olympus APX100 system.

### Cell sorting.

For flow sorting, we followed methods described by Herms et al. (75). Briefly, cells were collected and washed with PBS. The cell density was adjusted to 1 × 10^7^ cells/mL, filtered with a cell strainer of 70 μm, and sorted by side scatter (SSC-A) values using a FACSAria Fusion (BD Biosciences), collecting the population in the extremes (high SSC and low SSC). After sorting, cells were stained with BODIPY to validate lipid droplets.

### shRNA transfection.

Inducible scrambled and DGAT1 shRNA plasmids were obtained from Biosettia and transformed into TOP 10 *E*. *coli*–competent cells (Tiangen). The positive clone was selected and subsequently grown in Luria-Bertani broth overnight. The PureYield Plasmid Maxiprep system (Promega) was used to extract the plasmid. HEK293T cells were transfected with DGAT1 or scrambled plasmid along with psPAX2 (Addgene) and pMD2.G (Addgene) using Lipofectamine 2000 (Invitrogen). Lentivirus was collected after 48, 72, and 96 hours. Stable cell lines were then transfected with the lentivirus, followed by blasticidin selection. Doxycycline was used to induce knockdown, which was confirmed by quantitative RT-PCR.

### Real-time PCR.

Expression of genes was determined using SsoAdvanced Universal SYBR Green Supermix (Bio-Rad) and ViiA 7 Real-Time PCR System (Applied Biosystems). RNA was isolated using an Aurum Total RNA Mini Kit (Bio-Rad), followed by cDNA preparation from total RNA using an iScript cDNA Synthesis Kit (Bio-Rad). Primer pairs used in quantitative real-time PCR experiments were DGAT1 forward 5′-TCGATGATGCGTGAGTAGTCC-3′ and reverse 5′-CAATCTGACCTACCGCGATCT-3′ and β-actin forward 5′-GGATCAGCAAGCAGGAGTATG-3′ and reverse 5′-AGAAAGGGTGTAACGCAACTAA-3′.

### Statistics.

A log-rank test was used for survival analyses. Statistical data analysis (1-way ANOVA followed by Tukey’s post hoc test, or 2-tailed Student’s *t* test) was performed using Origin Pro software (Origin Lab Corp.). Unless otherwise indicated, data are presented as mean ± SEM.

### Study approval.

This study did not involve any human participants. All animal studies were performed in an Association for Assessment and Accreditation of Laboratory Animal Care International–accredited facility at Corewell Health Research Institute. All animal experiments were approved by the Institutional Animal Care and Use Committee at Corewell Health Research Institute and were performed in accordance with animal protocols AL-18-10, AL-19-07, and AL-2021-04. All in vivo experiments were conducted in compliance with institutional guidelines and approved by the Institutional Animal Care and Use Committee of Corewell Health William Beaumont University Hospital.

### Data availability.

The Supporting Data Values file contains all the underlying values for data presented in this article.

## Author contributions

SK and PC conceptualized the study. SK, YZ, KA, GM, NA, SFG, and PC developed methodology. SK, YZ, PK, KA, GM, and NA acquired data. SK, YZ, PK, KA, GM, and NA conducted experiments. SK, YZ, PK, KA, JFO, GM, NA, SFG, and PC performed formal analysis. SFG, CRM, and PC provided resources. SK, YZ, KA, JFO, GM, NA, SFG, CRM, and PC wrote the manuscript. PC acquired funding. SK and YZ contributed equally as co–first authors; their order is alphabetical. All authors have read and agreed to the published version of the article.

## Funding support

This work is the result of NIH funding, in whole or in part, and is subject to the NIH Public Access Policy. Through acceptance of this federal funding, the NIH has been given a right to make the work publicly available in PubMed Central.

- NIH/National Institute of Neurological Disorders and Stroke grants R01NS110838 and R01NS129744.

## Supplementary Material

Supplemental data

Unedited blot and gel images

Supplemental table 1

Supporting data values

## Version 1 12/09/2025

In-Press Preview

## Version 2 01/23/2026

Electronic publication
