# Supplementary material for: Enhanced lipid metabolism serves as a metabolic vulnerability to polyunsaturated fatty acids in glioblastoma
Source: JCI Insight. 2025 Dec 9;11(2):e191465. doi: 10.1172/jci.insight.191465 (PMC12892916; doi:10.1172/jci.insight.191465)
Supplement: Supplemental data [file jciinsight-11-191465-s280.pdf]

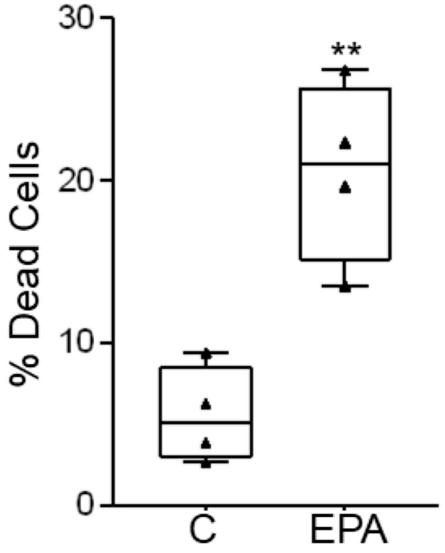

Supplementary Figure 1. Evaluation of the generalizability of PUFA-induced cytotoxicity in GBM. MES83 cells were treated with eicosapentaenoic acid (EPA) or vehicle control (C), and cell death was quantified by trypan blue exclusion assay. A significant increase in cell death was observed following EPA treatment **(**p < 0.01),** validating the anti-tumor activity of polyunsaturated fatty acids in GBM

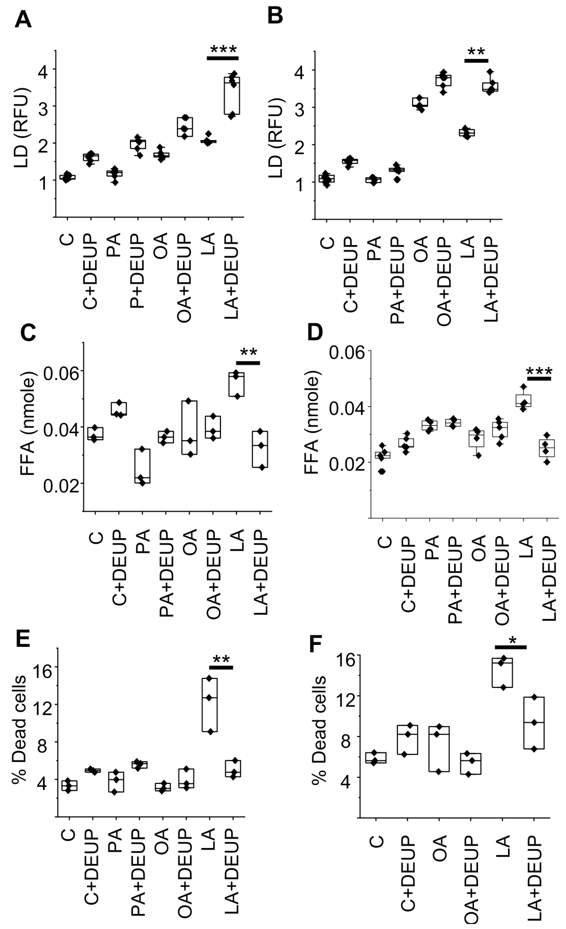

Supplementary Figure 2. The polyunsaturated fatty acid linoleic acid modulates lipid droplet dynamics by activating lipase activity in GBM. MES83 (**A,C,E**) and U251 (**B,D,F**) cells were pretreated with +/- diethylumbelliferyl phosphate DEUP (100uM, 45 min) followed by treatment with indicated fatty acids (palmitate [PA], oleic acid [OA], linoleic acid [LA]; 200μM). Lipid droplets (**A,B**; LD) and free fatty acids (**C,D**; FFA) were measured after 24h of the treatment. Non-viable cells (**E,F**) were counted with trypan blue after 72 h. Boxes represent the interquartile range, median and whiskers denote the upper and lower limit. $*p < 0.05$; $**p < 0.005$; $***p < 0.0005$. RFU: Relative fluorescence unit. S2A and S2B data without Fatty acids inhibitor are duplicated of 3J and 3L.

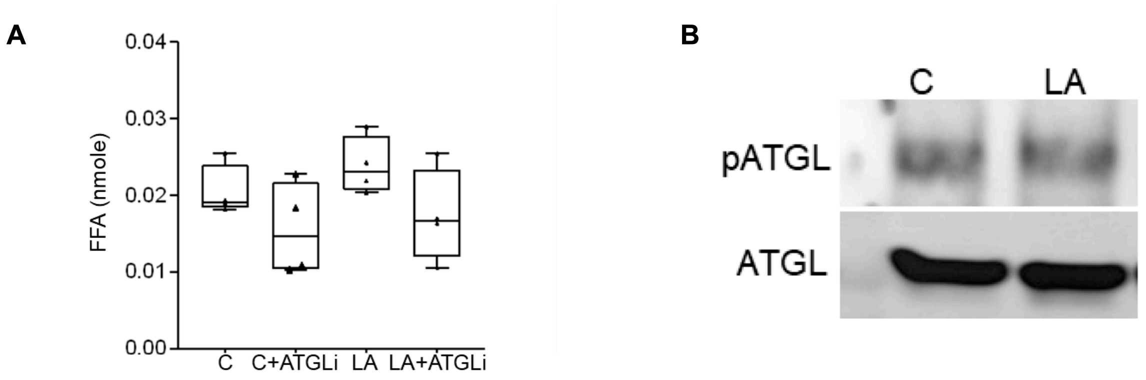

Supplementary Figure 3. PUFA treatment does not alter lipid metabolism or ATGL activation in proneural GBM cells. PN19 cells were treated with linoleic acid (LA; 200 μM) for 24 h and evaluated for free fatty acid (FFA) accumulation and ATGL phosphorylation. No significant changes were observed in FFA, or pATGL levels, consistent with the absence of enhanced lipid metabolism in proneural GBM.

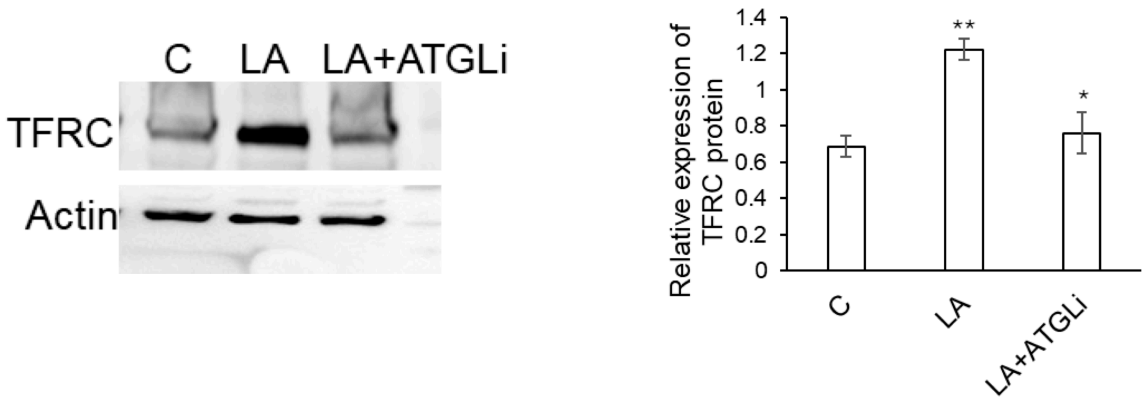

Supplementary Figure 4. Inhibition of ATGL rescues GBM cells from PUFA-induced ferroptosis. MES83 cells were treated with linoleic acid (LA; 200 μM) with or without the ATGL inhibitor atglistatin (ATGLi: 25 μM). Transferrin receptor (TFRC) expression were quantified. Atglistatin rescued GBM cells from LA-induced ferroptosis. *$p<0.05$, **$p<0.01$, significantly different from their respective controls.

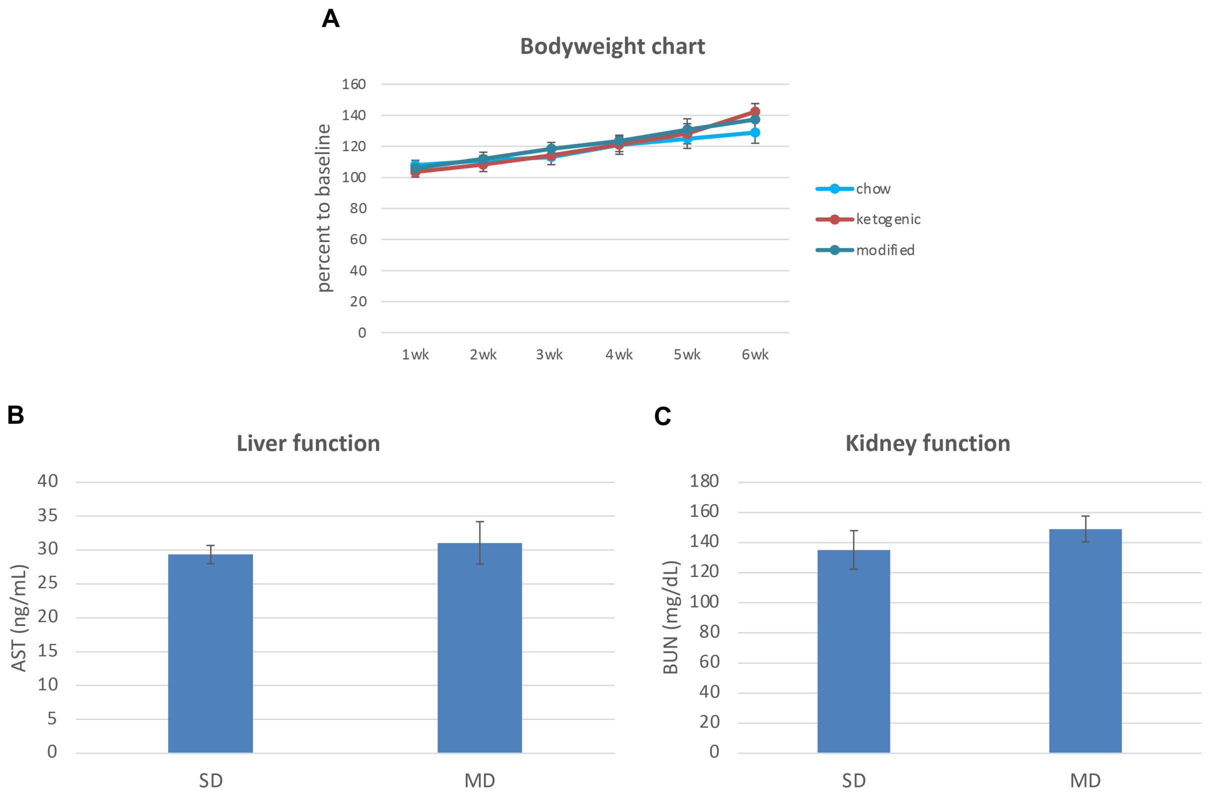

Supplementary Figure 5. Assessment of tolerability of the PUFA-rich modified diet in vivo. (A). Bodyweight measurements of mice fed the PUFA-rich modified diet compared to standard diet (chow) and ketogenic diet over the course of the study. No significant changes in bodyweight were observed between groups. (B and C). No significant differences in aspartate aminotransferase (AST) or blood urea nitrogen (BUN) were observed between dietary groups, showing the absence of ketogenic diet-induced hepatic or renal toxicity.
