# Supplementary material for: Enhanced lipid metabolism serves as a metabolic vulnerability to polyunsaturated fatty acids in glioblastoma
Source: JCI Insight. 2025 Dec 9;11(2):e191465. doi: 10.1172/jci.insight.191465 (PMC12892916; doi:10.1172/jci.insight.191465)
Supplement: Unedited blot and gel images [file jciinsight-11-191465-s281.pdf]

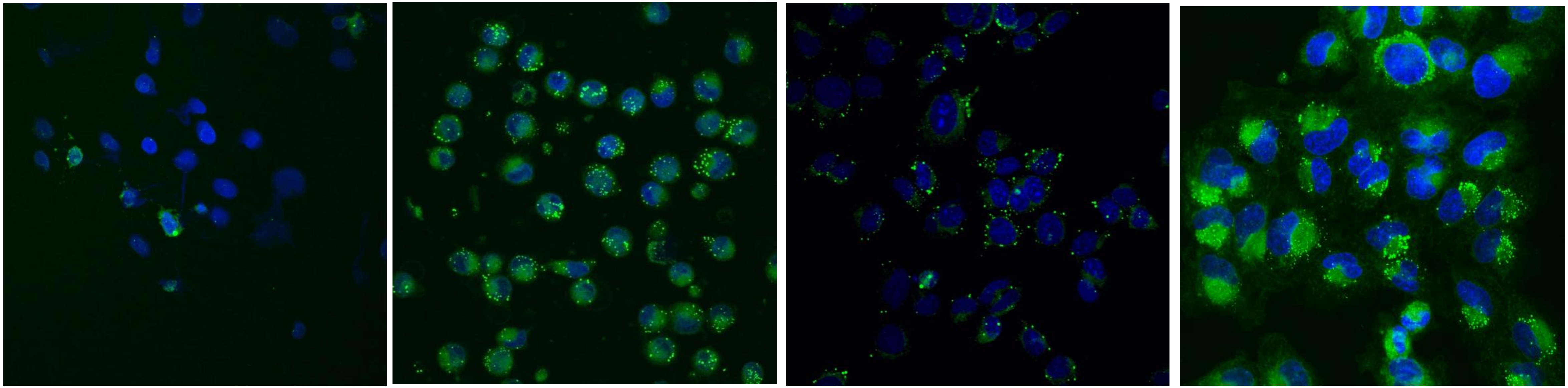

PN19 MES83 TRP U251

Unedited images for Figure 3B

U251

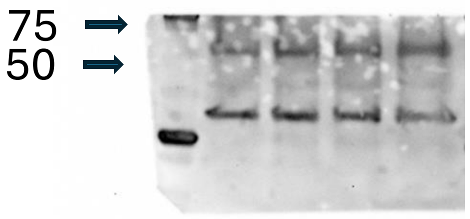

ATGL

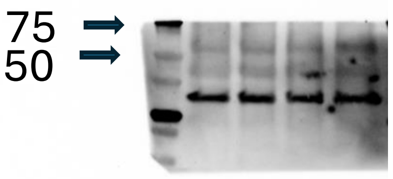

pATGL

Full unedited blot/gel for Figure 4J

TRP

75
50

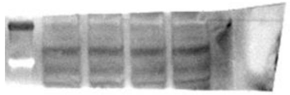

pATGL

75
50

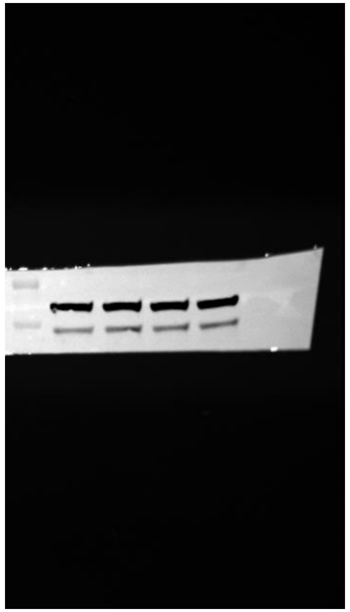

ATGL

Full unedited blot/gel for Figure 4K

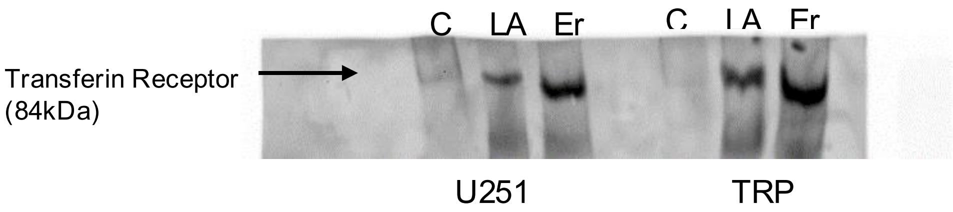

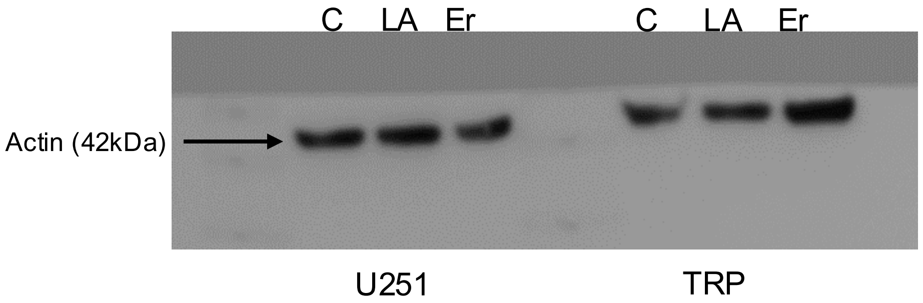

Full unedited blot/gel for Figure 5D

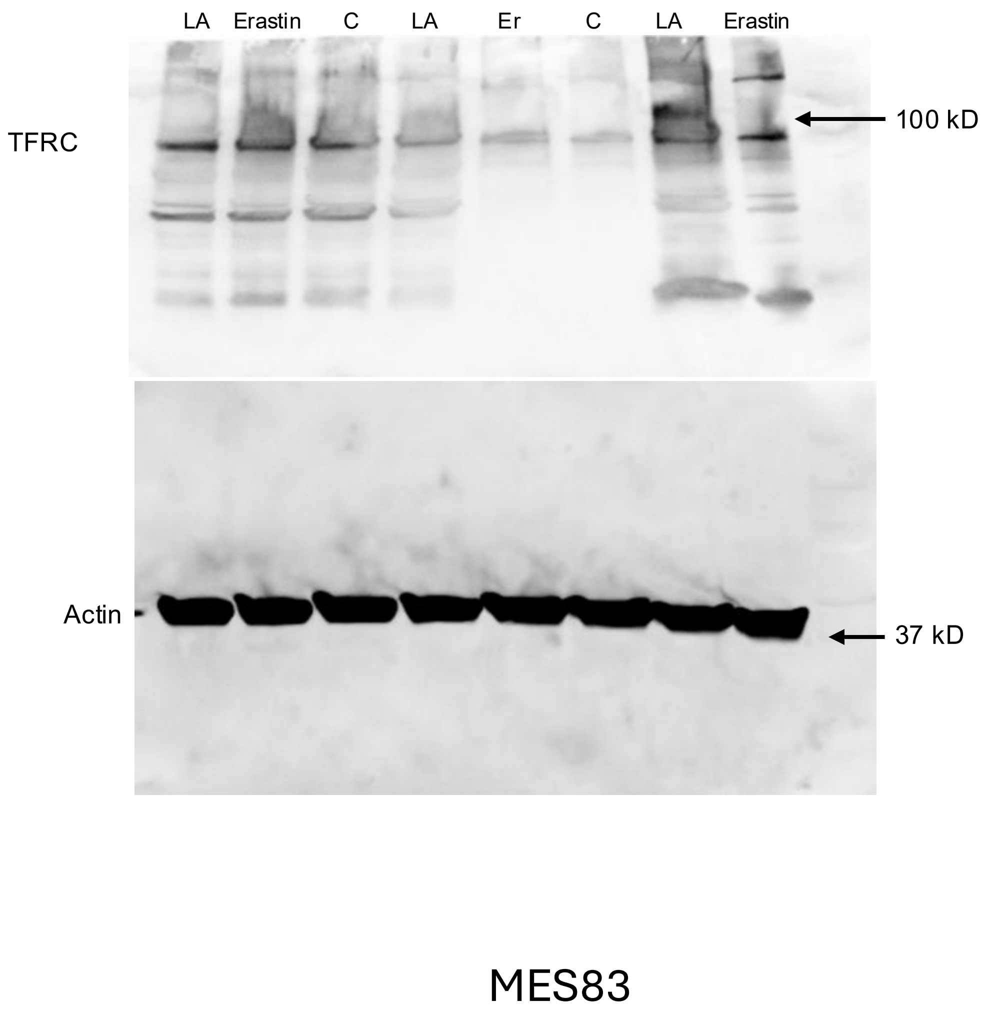

Full unedited blot/gel for Figure 5D

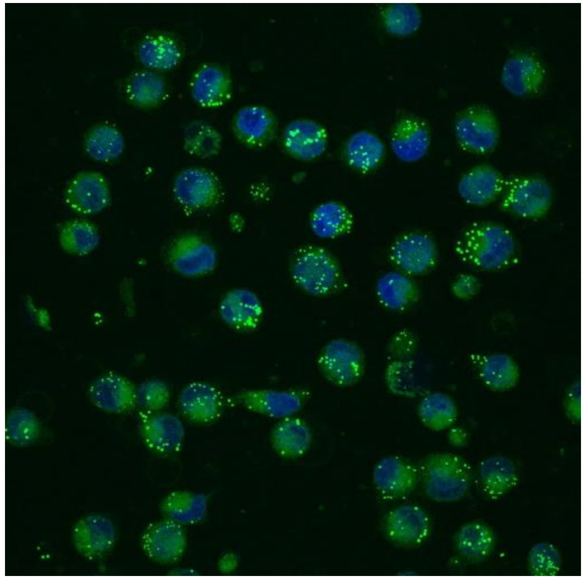

6B

6C

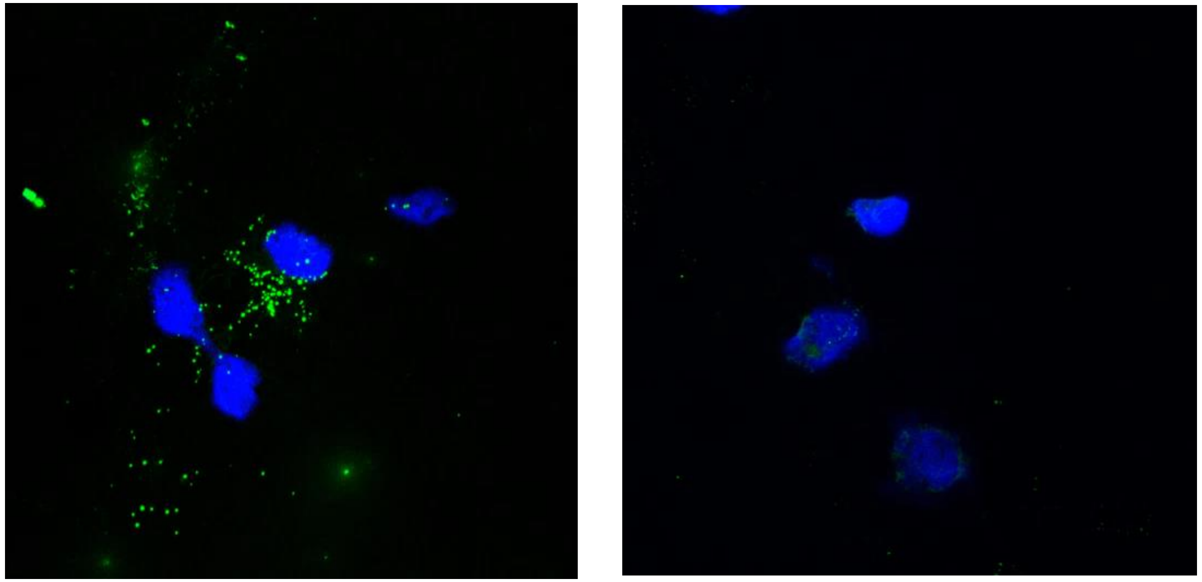

Unedited images for Figures 6B and 6

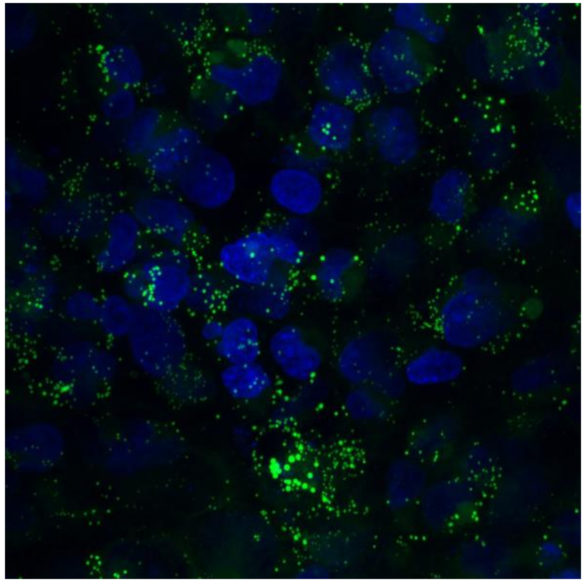

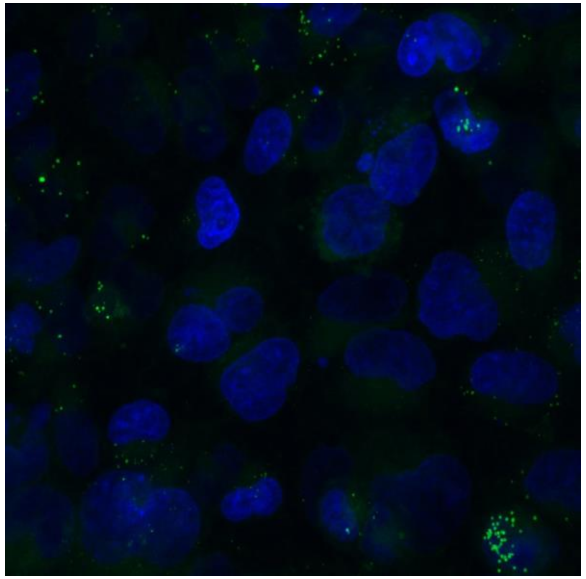

Unedited images for Figure 7E

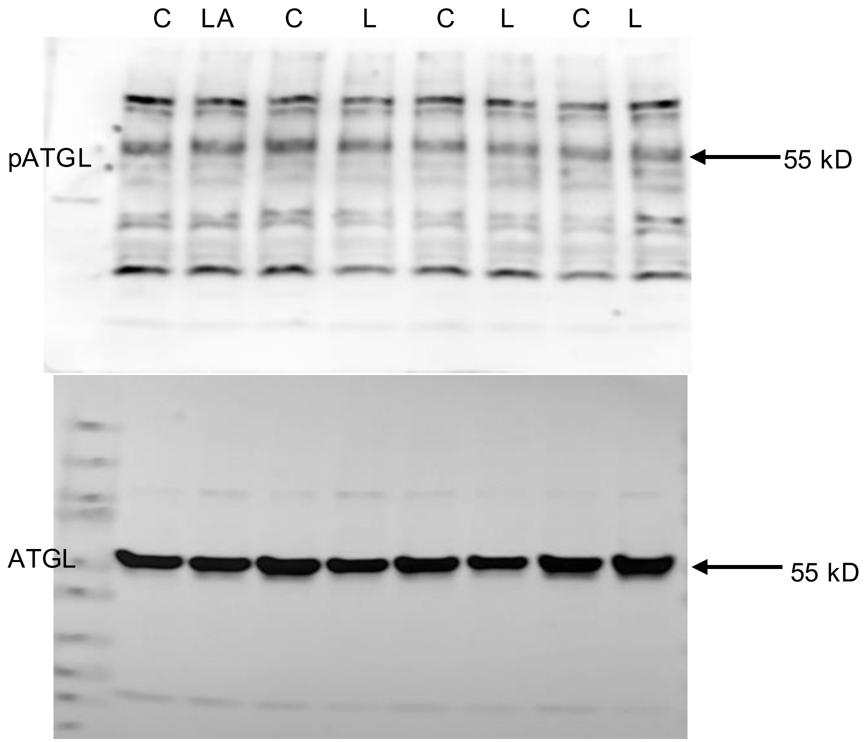

Full unedited blot/gel for Supplementary Figure 3

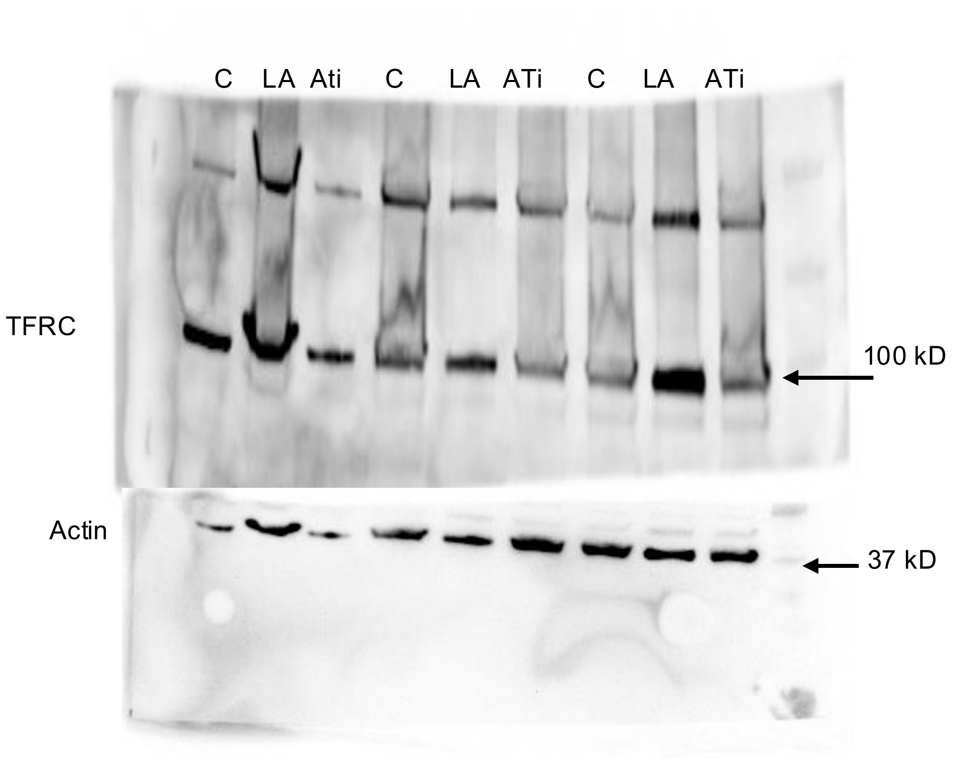

Full unedited blot/gel for Supplementary Figure 4
